# A comparison of emergency and therapeutic modified Shirodkar cerclage: an analysis of 38 consecutive cases

**DOI:** 10.4274/tjod.galenos.2018.33410

**Published:** 2019-03-27

**Authors:** Alper Başbuğ, Ozan Doğan

**Affiliations:** 1 Düzce University Faculty of Medicine Hospital, Clinic of Obstetrics and Gynecology, Düzce, Turkey; 2 University of Health Sciences, İstanbul Şisli Hamidiye Etfal Training and Research Hospital, Clinic of Obstetrics and Gynecology, İstanbul, Turkey

**Keywords:** Premature birth, cervical incompetence, cervical cerclage

## Abstract

**Objective:**

To compare the maternal and neonatal outcomes of patients with emergency versus therapeutic cerclage.

**Materials and Methods:**

The study included 38 female patients who underwent cervical cerclage using the modified Shirodkar method in the Obstetrics and Gynecology Clinics of Düzce University Medical Faculty Hospital and Düzce Atatürk State Hospital.

**Results:**

The operating time for the emergency cerclage group was significantly longer than that of the therapeutic group (30.40 minutes vs 19.85 minutes, p=0.001). Following the cerclage procedure, the cervical length was longer in the therapeutic cerclage group [29.90 millimeters (mm) vs. 22.45 mm, p=0.001]. The cerclage to birth interval was also longer in the therapeutic group (91 vs. 138 days).

**Conclusion:**

In comparison with therapeutic cerclage, the total duration of pregnancy after emergency cerclage is shorter, and newborns have a greater need for intensive care. Both methods, however, protect against advanced prematurity, which causes neonatal loss.

**PRECIS:** Both cerclage methods administered by experienced surgeons in appropriate patients could be effective.

## Introduction

Preterm labor and the resulting preterm births remain a major problem for physicians, and despite all the advances made, it remains the leading health problem in the field of obstetrics in both developed and developing countries^(1,2)^. There is a negative correlation between gestational age at birth and mortality and morbidity, primarily due to respiratory distress syndrome, intraventricular bleeding, necrotizing enterocolitis, and sepsis^(3,4)^. Several factors are responsible for the etiology of preterm labor^(5)^. One of these is cervical incompetence, which is seen in two percent of all pregnancies, and which leads to miscarriage in the second trimester and early third trimester^(6,7)^.

During the last 50 years, cervical cerclage has been frequently used to prolong the period until birth in pregnancies with cervical incompetence. In patients with cervical dilatation with or without prolapse of the amniotic membranes into the vagina, the procedure is conducted as an emergency cerclage. If cervical shortening is seen on serial ultrasonography measurements, the procedure can be conducted as a therapeutic cerclage^(8,9)^. The cervical cerclage procedure can be performed transvaginally with the McDonald and Shirodkar methods or transabdominally^(10,11,12)^. The efficacy of these methods in therapeutic and emergency cerclage is controversial, but the McDonald type of cerclage is more widely used because the application is easier to perform^(13,14)^.

The aim of this study was to evaluate the maternal and neonatal outcomes when modified Shirodkar cervical cerclage was performed as an emergency or a therapeutic procedure in a three-year period in two clinics.

## Materials and Methods

This retrospective case-control study included 38 pregnant women who were diagnosed as having cervical incompetence and treated with cervical cerclage using the modified Shirodkar method. The patients were treated in the Obstetrics and Gynecology Clinics of Düzce University Medical Faculty Hospital (a tertiary hospital) and Düzce Atatürk State Hospital (a secondary hospital) between June 2015 and May 2018.

Approval for the study was granted by the Institutional Ethics Committee, and all procedures were performed in accordance with the 1964 Helsinki Declaration. Informed consent was obtained from all study participants. This study followed the Strengthening the Reporting of Observational Studies in Epidemiology (STROBE) guidelines.

The therapeutic cerclage procedure was administered to patients with a cervical length of less than 25 mm without cervical dilatation in the second trimester sonographic cervical examination. The emergency cerclage procedure was performed on those with cervical dilatation determined during physical examinations and/or prolapse of the fetal membranes into the vagina.

Patients with symptoms of clinical and biochemical chorioamnionitis [tenderness of the cervix and/or the uterus, a temperature greater than 38° Celsius, a white blood cell count greater than 15.000 per cubic millimeter (mm^3^), and a C-reactive protein level greater than 2.0 milligrams per deciliter (mg/dL)], as well as those who had preterm premature rupture of membranes, a fetus with anomalies, a history of cone biopsy and loop electrosurgical excision procedures, mechanical dilation of the cervix during pregnancy termination, and uterine malformations were excluded from the study. A 12-hour waiting period was imposed before performing cerclage in order to exclude patients with preterm deliveries.

### Modified Shirodkar cerclage procedure

All procedures were conducted under regional or general anesthesia with the patient in the dorsal lithotomy position. A non-absorbable monofilament suture of 0.5 centimeters (cm) in thickness and 50 cm in length was used as the suture material, with a double needle for application (Braun, Aesculap, Tuttlingen, Germany). Before the therapeutic procedure, the cervix was visualized using a Sims retractor, and vaginal lavage was applied with a diluted iodine solution. Then, holding the anterior lip of the cervix with an Allis clamp, the physician performed an anterior colpotomy by making a 3 cm transverse incision into the anterior wall of the vagina after pulling it downward. The bladder was avoided. From the twelve o’clock position at the level of the cardinal ligament, 3-4 cm from the cervical os, the two-way needle with the suture material was passed to the level of four o’clock and eight o’clock. Then, removing both needles at the six o’clock position, the physician knotted the suture at the posterior of the cervix. The incision in the vaginal anterior wall was closed with a non-absorbable 3/0 multifilament suture. In the emergency cerclage procedure, all steps were applied in the same way, and the amniotic membrane that was prolapsed into the vagina was gently replaced into the uterus using wet sponges.

All operations were performed by A.B. and O.D., who are both experienced in these procedures.

The conventional Shirodkar suture was modified to decrease the operation time and to minimize the potential harmful effects of the anesthetic on the fetus, as well as to avoid the occurrence of hemorrhaging during the posterior vaginal wall dissection. Moreover, the modification simplifies the removal of the cerclage suture upon the commencement of labor.

After the procedure, all patients were observed for the following two days in the obstetric unit.

Prophylaxis of 1 gram (g) cefazolin sodium (Sefazol, M. Mevzat, Turkey) was applied before all the cerclage procedures. To inhibit uterine contractions, 100 mg of indomethacine (Endol, Deva, Turkey) was administered rectally. For the relief of postoperative pain, 1 g of acetaminophen (Parol, Atabay, Turkey) was administered. Additionally, in the emergency cerclage group, all patients were given 1 g of cefazolin sodium intravenously twice a day and 500 mg of metronidazole intravenously twice a day for five days. All patients in both groups received 200 mg of natural progesterone vaginally for as long as their pregnancies continued or until the 37^th^ gestational week. Tocolysis with oral nifedipine was implemented on an individual basis.

The presence of uterine contractions that did not respond to tocolysis and the rupture of the membranes were regarded as indications for preterm removal of the cerclages. Otherwise, the cerclages were electively removed at 37 gestational weeks.

The primary goal was to evaluate the relationship between maternal and perinatal outcomes and the cerclage procedure type. The secondary goal was assessing the relationship between cervical dilatation, residual cervical length and vaginal contact with the amniotic membranes, and births before the 32^nd^ gestational week.

### Statistical Analysis

Analysis of the data obtained in the study was made using the SPSS (Ver. 22.0) software. Quantitative data are presented in the tables as mean ± standard deviation and median (minimum-maximum) values. Categorical data are stated as number (n) and percentage (%). In the comparison between independent groups, Student’s t-test and the Mann-Whitney U test were used, and in the comparison of categorical variables, Pearson’s chi-square test and Fisher’s exact test were used. Univariate binary logistic regression analysis was used to determine relationships between risk factors that were independently associated with delivery at less than 32 gestational weeks. No power calculation was performed for this study because all eligible patients were included in the study. The data were examined at 95% confidence intervals (CI). A p value of <0.05 was accepted as being statistically significant.

## Results

The study included a total of 38 patients, comprising 22 emergency cases and 16 therapeutic cases. No statistically significant difference was determined between the groups with respect to demographic characteristics, premature birth history, the manner of pregnancy (natural or *in vitro *fertilization) or multiple pregnancies (Table 1).

The operating time was determined to be statistically significantly longer in the emergency cerclage group than in the therapeutic group (30.40 minutes vs 19.85 minutes, p=0.001). The cervical length after the cerclage procedure was determined to be statistically significantly shorter in the emergency group (20.24±3.97 mm) compared with the therapeutic group (29.90±4.65 mm) (p=0.001). During the procedure, membrane rupture was observed in two patients (9.1%) in the emergency group, and no membrane rupture associated with the procedure was seen in any of the therapeutic cerclage group. Following the procedure, chorioamnionitis was seen in one patient (4.5%) in the emergency cerclage group and in no patients in the therapeutic group. The cerclage procedure was applied at an earlier gestational week in the therapeutic group than in the emergency group, but the difference was not statistically significant (19 weeks vs 21 weeks, p=0.59). The time period from the procedure to birth was longer, and the gestational week was later in the therapeutic group than in the emergency group (128 days vs 80 days, p=0.001; 37 weeks vs 32 weeks, p=0.001).

Birth occurred at full-term (≥37 weeks) in 10 patients (62.5%) in the therapeutic cerclage group and in three patients (13.6%) in the emergency group. The difference between the groups was determined to be statistically significant (p=0.002). The rates of vaginal delivery and caesarean section were similar in both groups (p=0.22, p=0.51, respectively).

There was a greater need for tocolytic therapy in the emergency cerclage group following the procedure (p=0.001). There was a greater need for neonatal intensive care in the emergency group (p=0.001). The neonatal mortality rate was not observed in the therapeutic cerclage group, and it was 9.1% (n=2) in the emergency cerclage group, with no statistically significant difference determined between the groups (Table 2).

The cervical length after cerclage that was shorter than 20 mm, the vaginal contact of amniotic membranes and multiple pregnancies were associated with preterm delivery before 32 gestational weeks [odds ratio (OR)=5.83, 95% CI: (1.20-28.36), p=0.018; OR=1.93, 95% CI:(1.37-2.72), p=0.025; OR=2.91, 95% CI:(1.88-4.61), p=0.021]. Cervical dilatation was not seen as a risk factor for preterm delivery before 32 gestational weeks (Table 3).

## Discussion

The data obtained in this study demonstrated that the cervical cerclage procedure (whether therapeutic or emergency in nature), conducted because of cervical incompetence in the second and early third trimesters, is effective in preventing extreme pre-term births. In the therapeutic cerclage group, the time from the procedure to birth was longer than when the procedure was performed under emergency conditions. Furthermore, the residual cervical length after the cerclage procedure was longer in the therapeutic group. With cervical lengths after cerclage that were shorter than 20 mm, vaginal contact with the amniotic membranes and multiple pregnancies were significantly more prevalent in patients who delivered preterm before 32 gestational weeks.

Berghella et al.^ (15)^ suggested that cervical lengths could be observed with transvaginal ultrasounds in women with a history of pre-term births because of cervical incompetence, and that emergency cerclage should be applied only if a short cervix was determined. In contrast, Guzman et al.^ (16)^ reported that better results were obtained with therapeutic cerclage as compared with emergency cerclage. In the current study, the cerclage to birth interval was more favorable in the therapeutic cerclage group. There was a lesser need for neonatal intensive care after birth in the therapeutic cerclage group, but there was no difference between the groups in terms of the neonatal mortality rate.

Therapeutic cerclage is generally applied early in the second trimester and is a relatively low risk operation. An emergency cerclage procedure is applied more typically in the middle of the second trimester, when the cervix is significantly shortened, when there is cervical dilatation and when membranes are prolapsed into the vagina; therefore, it is an operation in which complications such as membrane rupture and chorioamnionitis are frequently seen^(17)^. In the current study, although the rates of membrane rupture associated with the procedure and chorioamnionitis after the procedure were higher in the emergency cerclage group, the difference between the groups was not statistically significant.

In a retrospective analysis of 158 patients treated with the McDonald method, Zhuve et al.^ (18)^ found that 10% of patients maintained the pregnancy beyond the 37^th^ gestational week. In a study by Ohad et al.^ (19)^, the results of patients having had the McDonald procedure as a therapeutic or an emergency procedure were compared, and 64% of the therapeutic group and 59% of the emergency group gave birth after the 37^th^ gestational week. In the current study, the rate of births in the 37^th^ gestational week or later was 62.5% in the therapeutic group and 13.6% in the emergency group. Ohad et al.^ (19)^ applied the emergency cerclage procedure to patients with cervical lengths of less than 25 mm in the second trimester. In the current study, all patients in the emergency cerclage group had cervical dilatation and/or prolapse of the amniotic membranes into the vagina. Contact between the fetal membranes and the vagina increases the risk of chorioamnionitis and intra-amniotic infections. These infections are sometimes clinically evident, and some continue as subclinical infections and trigger pre-term labor^(18,20,21)^. This can be considered to be the reason for the high rate of pre-term births in the current study’s emergency cerclage group. At the same time, when the cervix has shortened to an advanced degree, and the amniotic membranes have prolapsed into the vagina, it is extremely difficult to place the cerclage suture at the level of the internal cervical os. Thus, it is clear that the efficacy of sutures that have not reached a sufficient length will be reduced. In comparison with the therapeutic group, the fact that sutures could not be placed in the appropriate position in the emergency cerclage group can be considered to be one of the factors that prevent the pregnancy from reaching full-term.

The cervical cerclage technique has been used for more than 50 years in the prevention of pre-term births associated with cervical incompetence^(22)^. However, very few studies have compared the efficacy of the application of different procedures. Obido et al.^ (23)^ found no difference between the McDonald and Shirodkar procedures in the prevention of pre-term births. In contrast, Treadwell et al.^(24)^, and more recently, Wong et al.^(25)^, reported that the Shirodkar procedure was more effective than the McDonald procedure in terms of preventing pre-term births. In a review published by Berghella^(26)^, it was stated that the residual cervical length after cerclage should be greater than 2 cm to be effective in the prevention of pre-term birth; therefore, the cerclage suture must be applied as close as possible to the internal cervical os. Accordingly, to get as close as possible to the cervical os, it is necessary to dissect the bladder. In this respect, the Shirodkar procedure has an advantage over the McDonald procedure in reaching a satisfactory cervical height. In the current study, the Shirodkar procedure was applied to all patients, whether therapeutic or emergency, and in comparison with the McDonald procedure, there was considered to be a greater contribution to residual cervical height. There was also better support of the cervical tissue.

## Conclusion

The strength of the study is that it is one of few studies that have compared the results of emergency and therapeutic cerclage procedures. Moreover, because the procedures were applied by surgeons experienced in this field, this standardized the applications. Basic limitations of the study include that it was retrospective, that there were relatively few patients, and that there was an insufficient number of vaginal cultures taken before the cerclage procedures.

In conclusion, despite the advances that have been made in obstetrics, pre-term births remain a major problem. In the prevention of pre-term birth as a result of cervical incompetence, both therapeutic and emergency cerclage procedures are effective. However, it must be noted that with regular screening of the cervical length from the second trimester onward in high-risk pregnancies, this procedure should be applied before cervical dilatation and prolapse of the amniotic membranes into the vagina.

## Figures and Tables

**Table 1:**
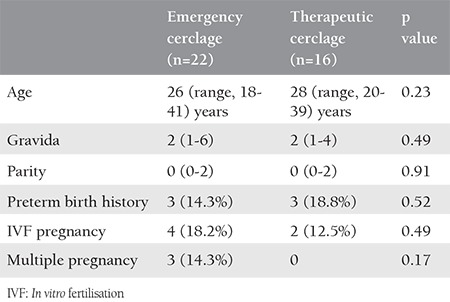
The demographic characteristics of patients who underwent emergency and therapeutic cerclage procedures

**Table 2:**
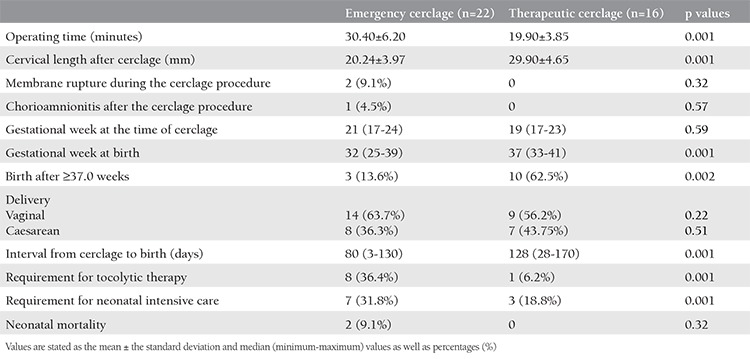
The operative and perinatal findings of patients who underwent emergency and therapeutic cerclage

**Table 3:**
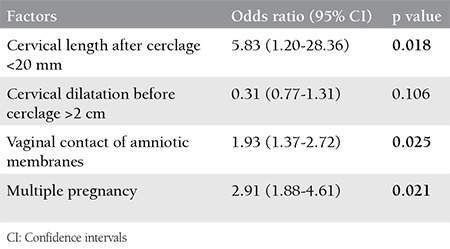
Univariate logistic regression analysis for the association between possible risk factors and delivery before 32 gestational weeks
